# Human stem cell-derived GABAergic neurons functionally integrate into human neuronal networks

**DOI:** 10.1038/s41598-021-01270-x

**Published:** 2021-11-11

**Authors:** Ana Gonzalez-Ramos, Eliška Waloschková, Apostolos Mikroulis, Zaal Kokaia, Johan Bengzon, Marco Ledri, My Andersson, Merab Kokaia

**Affiliations:** 1 Epilepsy Center, Department of Clinical Sciences, Lund University Hospital, 22184 Lund, Sweden; 2 Lund Stem Cell Center, Department of Clinical Sciences, Lund University Hospital, 22184 Lund, Sweden; 3 Department of Clinical Sciences, Lund University, Skånes Universitetssjukhus, 22184 Lund, Sweden

**Keywords:** Synaptic transmission, Stem-cell research, Translational research

## Abstract

Gamma-aminobutyric acid (GABA)-releasing interneurons modulate neuronal network activity in the brain by inhibiting other neurons. The alteration or absence of these cells disrupts the balance between excitatory and inhibitory processes, leading to neurological disorders such as epilepsy. In this regard, cell-based therapy may be an alternative therapeutic approach. We generated light-sensitive human embryonic stem cell (hESC)-derived GABAergic interneurons (hdIN) and tested their functionality. After 35 days in vitro (DIV), hdINs showed electrophysiological properties and spontaneous synaptic currents comparable to mature neurons. In co-culture with human cortical neurons and after transplantation (AT) into human brain tissue resected from patients with drug-resistant epilepsy, light-activated channelrhodopsin-2 (ChR2) expressing hdINs induced postsynaptic currents in human neurons, strongly suggesting functional efferent synapse formation. These results provide a proof-of-concept that hESC-derived neurons can integrate and modulate the activity of a human host neuronal network. Therefore, this study supports the possibility of precise temporal control of network excitability by transplantation of light-sensitive interneurons.

## Introduction

GABA-releasing interneurons comprise a highly abundant cell type in the central nervous system. Although they represent a minority of the total neuronal population (only 20% in comparison to 80% of the excitatory neurons), they exert a strong inhibitory effect on principal glutamatergic neurons, controlling network excitability. Furthermore, interneurons modulate cortical maturation, synchronous network oscillations and network plasticity^1,2^. GABAergic interneurons are highly heterogeneous, forming different subpopulations based on their function, morphology and connectivity^3–5^. Dysfunction of interneurons has been implicated in neurological disorders, including schizophrenia, autism, and epilepsy^6,7^. For instance, in temporal lobe epilepsy, the dysfunction and decreased numbers of interneurons in the hippocampus leads to a disruption of the normal hippocampal circuitry resulting in a hyperexcitable neuronal network and seizures^8^.

Human pluripotent stem cells are a powerful tool for both modelling brain development and disease, as well as development of cell therapies. The possibility to differentiate patient-specific stem cells to mature regional- and transmitter- specific subtypes of particular human interneuron populations provides an exceptional platform for studying pathophysiology as well as a potential therapeutic approach for diseases. To this goal, several studies have focused on generating GABAergic neurons from human stem cells (hSC), both induced pluripotent stem cells and ESCs^9–11^. Moreover, due to the limited endogenous regeneration capacity of the human brain, transplantation of neural SCs or hSC-derived neurons into a diseased or injured brain is a promising therapeutic approach to restore neuronal population and function. In the past, several studies have shown that transplanted fetal rodent medial ganglionic eminence (MGE)-derived GABAergic progenitor cells can integrate into host tissue and restore function of lost interneurons in animal models of epilepsy^12,13^. Despite the high value of these studies, translation to the clinic requires a human cell source, generating a robust and consistent yield of GABAergic neurons, and proof of functional cell integration. In this regard, hSCs offer great potential as an unlimited source of derived neurons for cell-based therapeutic strategies.

In this study, we used a reprogramming approach based on the transgene expression of transcription factors *Ascl1* and *Dlx2* to induce the differentiation of hESCs into a pure population of interneurons in a short time^14^. The functional maturation of the derived neurons during the differentiation process was also assessed electrophysiologically at various time points. Furthermore, hESC were genetically modified using optogenetics to permit modulation of activity of hdINs by light. Using optogenetics, we demonstrate for the first time that hdINs can integrate into a human neuronal network by forming functional efferent synapses onto human primary neurons. We further confirm integration of hdINs in human epileptic brain slices obtained from surgeries for drug-resistant epilepsy, and thereby provide opportunity to modulate disease-altered human neuronal networks.

## Results

### Human ESC-derived neurons exhibit a GABAergic phenotype and express calretinin and calbindin markers

**Figure 1:**
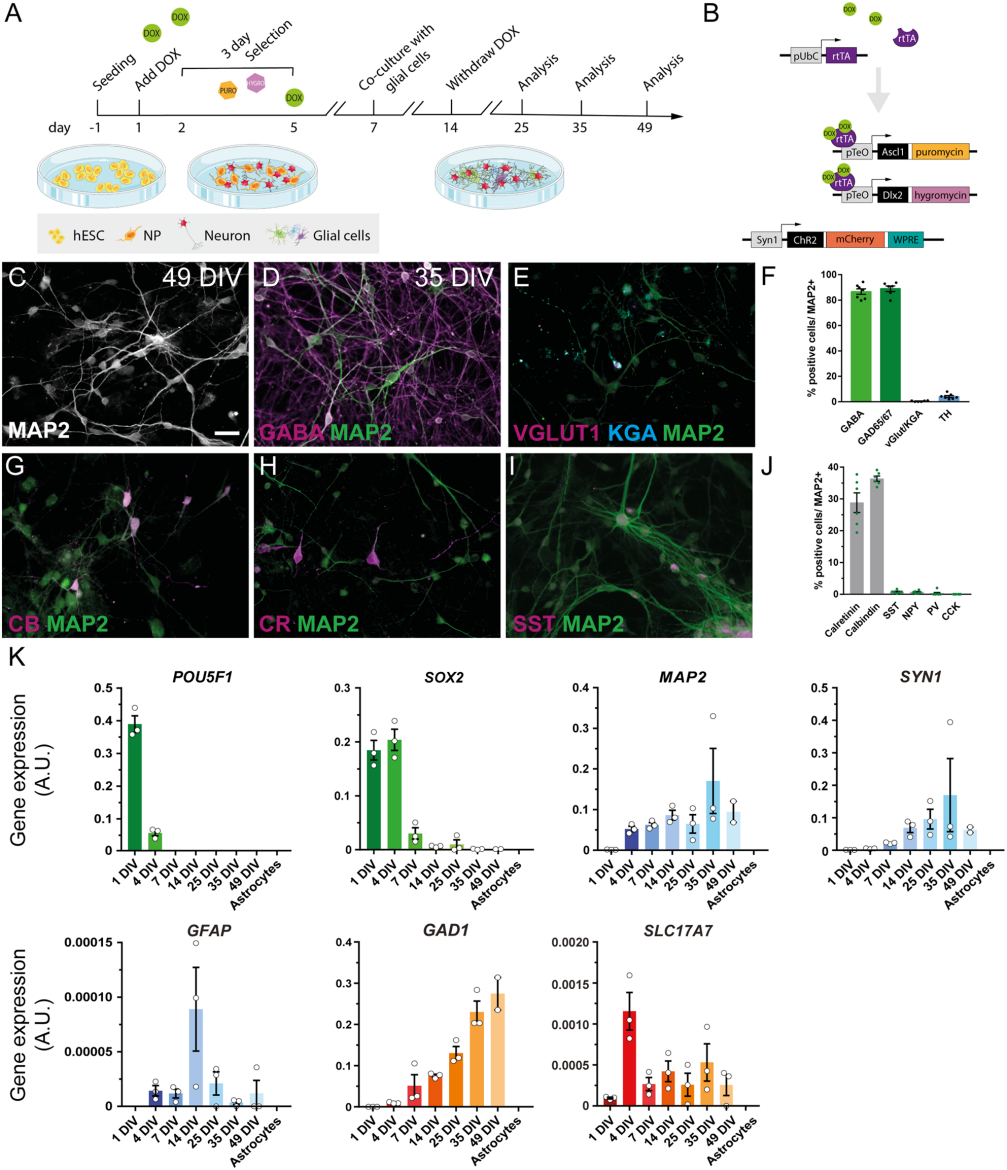
Transgene expression of the transcription factors *Ascl1* and *Dlx2* by Tet-On system triggers the differentiation of hESC to GABAergic neurons. (**A**) Differentiation protocol used for the generation of hdINs. (**B**) Schematic view of the constructs carried by the lentiviral particles used in the differentiation protocol (Tet-On system), and for the expression of ChR2. (**C**) Immunocytochemistry for the neuronal marker MAP2 + of the derived neurons at 49 DIV. (**D**, **E**) Immunocytochemistry of hdINs at 35 DIV for GABAergic (GABA) and glutamatergic (VGLUT1 and KGA) markers. (**F**) Quantification of the percentage of GABA +, GAD65/67 +, vGlut1 +/KGA + and TH + cells over MAP2 + cells as an average of both time points, 35 and 49 DIV. (**G**–**I**) Representative images of hdINs showing the presence of the interneuron markers CB, CR, and SST. (**J**) Quantification of the percentage of CR +, CB +, SST +, NPY +, PV + and CCK + subtypes over MAP2 + cells. (**K**) Gene expression profile during the differentiation. Values for the pluripotency gene *POU5F1* that encodes for OCT4, and *SOX2* gene in green. Genes expressed in mature cell populations such as *MAP2* and *SYN1* in neurons, and human *GFAP* in astrocytes where also analyzed and indicated in blue. Key genes of the two main neuronal populations in the brain were also studied and shown in orange, quantifying *GAD1* gene which is expressed in GABAergic neurons and *SLC17A7* gene which encodes for VGLUT1 and it is expressed in glutamatergic neurons. NP, neural precursor. Scale bar: 100 µm. Mean ± SEM. Schematics were generated and adapted using resources from Servier Medical Art^35^.

Previous studies have demonstrated the possibility to generate highly enriched GABAergic neuronal cultures from hESCs^9–11,14^. Here, hESCs were differentiated into GABAergic neurons using a single-step method overexpressing the transcription factors *Ascl1* and *Dlx2*, which are key factors for this lineage determination (Fig. 1A, B)^14^. The phenotype and electrophysiological properties of the hdINs were assessed at 25, 35, and 49 DIV (Fig. 1A). First, a clear gradual change in morphology was observed in the cell cultures during differentiation, accompanied by a change in gene expression, displayed by a reduction of the pluripotency marker OCT4, encoded by *POU5F1* gene, and the pluripotency and neural precursor marker *SOX2* (Fig. 1K). *POU5F1* had a maximal expression level at 1 DIV, which was decreased at 4 DIV, until not being detected from 7 DIV onwards. *SOX2* gene expression was high at 1 and 4 DIV with a marked reduction from the 7 DIV time point. On the other hand, the *MAP2* gene (neuronal marker) expression was already detected at 4 DIV and showed a tendency to increase its expression over time (Fig. 1C and K). Similarly, *SYN1* displayed a gradual increase of expression from 7 to 35 DIV, at which time point the maximal expression was reached indicating the onset of synaptic maturation of differentiated neurons (Fig. 1K). The slight reduction of *SYN1* expression at 49 DIV could be explained by the decrease of cell density due to reduced viability of astrocytes after long-term culturing in media containing Ara-C. Altogether, these changes indicated a fast, transitional dynamic switch from the expression of pluripotency towards neuronal markers around 4 and 7 DIV. Importantly, the differentiation protocol did not generate astrocytes or oligodendrocyte precursors from hESCs since human *GFAP* and human *PDGFRα* expression levels were hardly detectable (Fig. 1K and Figure S1).

Within the neuronal population, most of the hdINs were positive for GABA, 86.66 ± 2.14% positive cells out of MAP2 + (85.48 ± 2.98% at 35 DIV and 88.23 ± 3.46% at 49 DIV) (Fig. 1D and F). The GABAergic identity of the neuronal population was confirmed by expression of GAD65/67, 88.95 ± 3.55% positive cells out of MAP2 + (89.87 ± 2.11% at 35 DIV and 88.03 ± 4.3% at 49 DIV) (Fig. 1F) and *GAD1* gene expression levels (which encodes for GAD67) that increased over the differentiation timeline (Fig. 1K). These results demonstrate a predominantly GABAergic phenotype of cells over glutamatergic (VGLUT1/KGA, 0.41 ± 0.19% positive cells out of MAP2 +, Fig. 1E and F) or dopaminergic ones (TH, 4.1 ± 0.78% positive cells out of MAP2 +, Fig. 1F). The *SLC17A7* gene, encoding for VGLUT1, was barely detectable by RT-qPCR (Fig. 1K). Furthermore, within the GABAergic neuronal population, the most abundant subtypes were calbindin (CB)- and calretinin (CR)-expressing interneurons, representing 36.38 ± 0.82% (36.23 ± 1.3% at 35 DIV and 35.25 ± 0.25% at 49 DIV) and 28.83 ± 3.08% (35.37 ± 1.3% at 35 DIV and 22.3 ± 1.73% at 49 DIV) respectively (Fig. 1G, H and J). Among other interneuron subtypes tested, there were cells found expressing somatostatin (SST, 0.79 ± 0.19%, Fig. 1I–J) and neuropeptide-Y (NPY, 0.71 ± 0.17%, Fig. 1J), while almost none of them were positive for either parvalbumin (PV, 0.28 ± 0.28%) or cholecystokinin (CCK, 0%) (Fig. 1J). Results from the immunostaining were supported by gene expression data, where also *VIP* and *NKX2.1* (MGE marker) gene expression were included (Figure S1). *NKX2.1* gene expression started appearing at 4 DIV maintaining stable levels and increasing slightly at 25 until 49 DIV. These results support the possibility of having some early-stage neurons in the cultures. Moreover, expression of *PPP1R1B* gene that encodes for DARPP-32, a marker for striatal medium spiny neurons, which are GABAergic projecting neurons, was not present at 35 DIV and 49 DIV (Figure S1).

### Maturation of intrinsic electrophysiological properties of hdINs over time in culture and functional confirmation of their GABAergic nature

**Figure 2:**
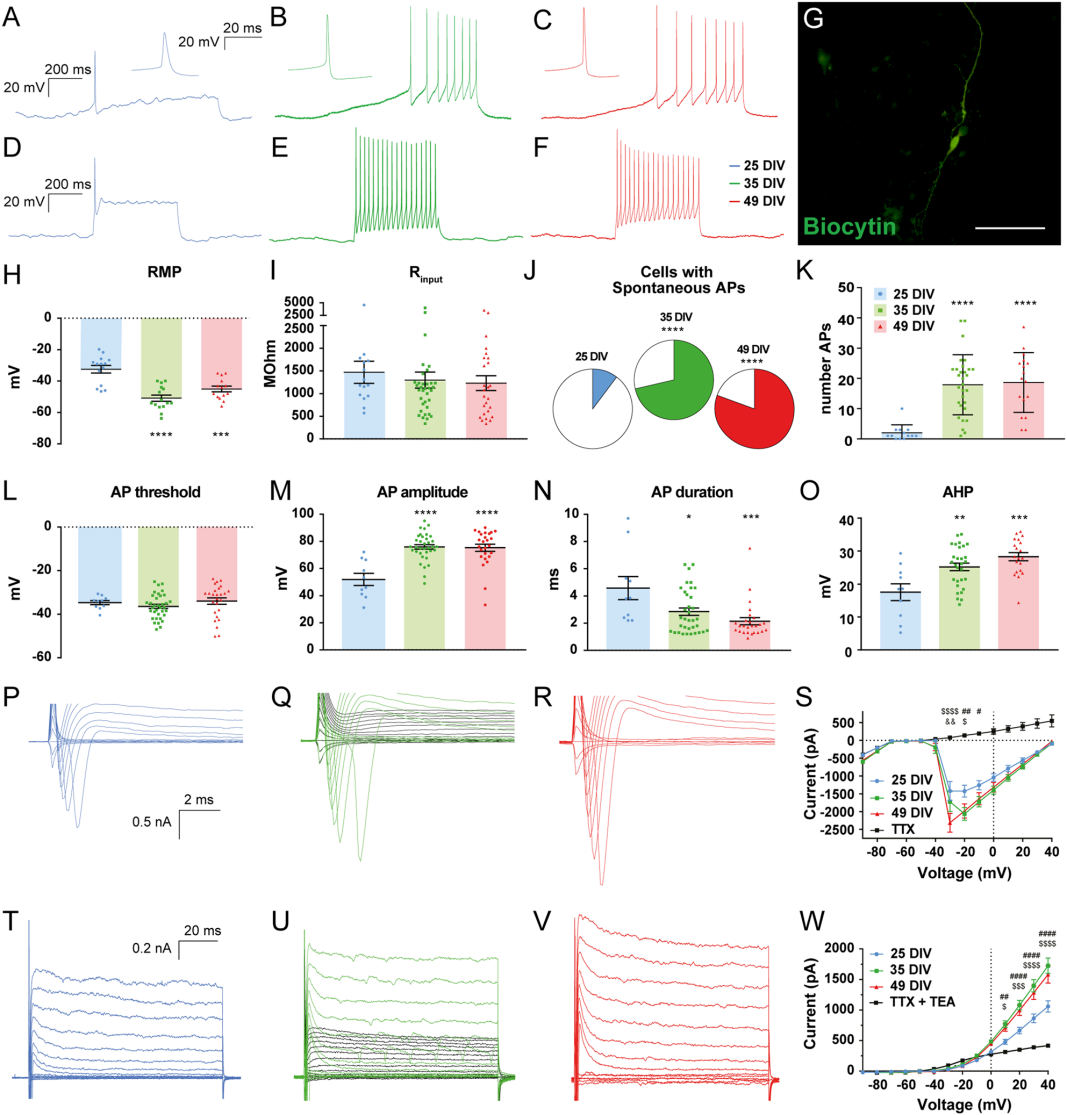
Electrophysiological properties of hdINs during maturation process over time in culture. Differential cell response (**A**–**C**) to 0–25 pA ramps of depolarizing current and (**D**–**F**) 50 pA depolarizing current pulses at different time points. (**G**) hdIN filled with biocytin. (**H**–**I**) RMP and Ri at different time points. (**J**) Increased proportion of cells with spontaneous APs (colored area) over time. (**K**) Maximum number of APs that cells fired in response to 500 ms current pulses. (**L**–**O**) Distribution of AP threshold (**L**), amplitude (**M**), duration (**N**) and afterhyperpolarization (**O**) at different time points. (**P**–**R**) Expanded current traces illustrating the sodium current and (**T**–**V**) the potassium current activated during voltage pulses ranging from − 90 to + 40 mV in 10 mV steps at different time points. Sodium and potassium currents were blocked by TTX (1 µM) and TTX + TEA (10 mM) respectively (black lines). Sodium (**S**) and potassium (**W**) current peak plotted against the voltage steps. Scale bar: 100 µm. Mean ± SEM. One-way ANOVA with Tukey’s post hoc test and Fisher’s exact test (25 DIV n = 15 in blue, 35 DIV n = 36 in green, and 49 DIV n = 26 in red). *p < .05; **p < .01; ***p < .001; ****p < .0001; compared to 25 DIV group. Two-way ANOVA with Tukey’s post-hoc test. #, 25DIV vs 35DIV; $, 25DIV vs 49DIV; &, 35DIV vs 49DIV (25 DIV n = 15 in blue, 35 DIV n = 28 in green, and 49 DIV n = 33 in red). AP, action potential; AHP, afterhyperpolarization; RMP, resting membrane potential; Ri, input resistance.

Electrophysiological properties of the hdINs at different time points were investigated to correlate functional maturation with the progress of their morphological changes during the differentiation process. Whole-cell patch-clamp recordings were performed at 25 DIV (blue), 35 DIV (green) and 49 DIV (red) (Fig. 2). Input resistance was similar at all time points analyzed (Fig. 2I and Table S1). Small differences in resting membrane potential in cells at both 35 and 49 DIV compared to 25 DIV were observed (− 34.27 ± 2.87 mV at 25 DIV, − 47.20 ± 1.84 mV at 35 DIV and − 44.85 ± 1.94 mV at 49 DIV) (Fig. 2H and Table S1). Already at 25 DIV, hdINs were able to fire multiple action potentials upon depolarization (Fig. 2K) and displayed both a fast-inward sodium ion current and a sustained outward potassium (K^+^) ion current (Fig. 2P–W). The percentage of hdINs firing to ramp depolarizing current was increased over time and action potentials displayed higher amplitudes, faster rise times and larger after-hyperpolarization at 35–49 DIV compared to 25 DIV (Fig. 2A–F and J–O, and Table S1). However, no statistically significant differences in these parameters were observed between the 35 DIV and the 49 DIV time points. These observed changes reflect an increase in functional voltage-dependent Na^+^ and K^+^ channels, supported by larger inward and outward current peaks at later time points (Fig. 2S and W). Altogether, these differences in intrinsic properties demonstrate a clear functional maturation of hdINs over time in culture, reaching a more mature state already at 35 DIV. The firing patterns observed were single, regular or clustered spiking, with no sustained spiking beyond 62 Hz observed.

**Figure 3:**
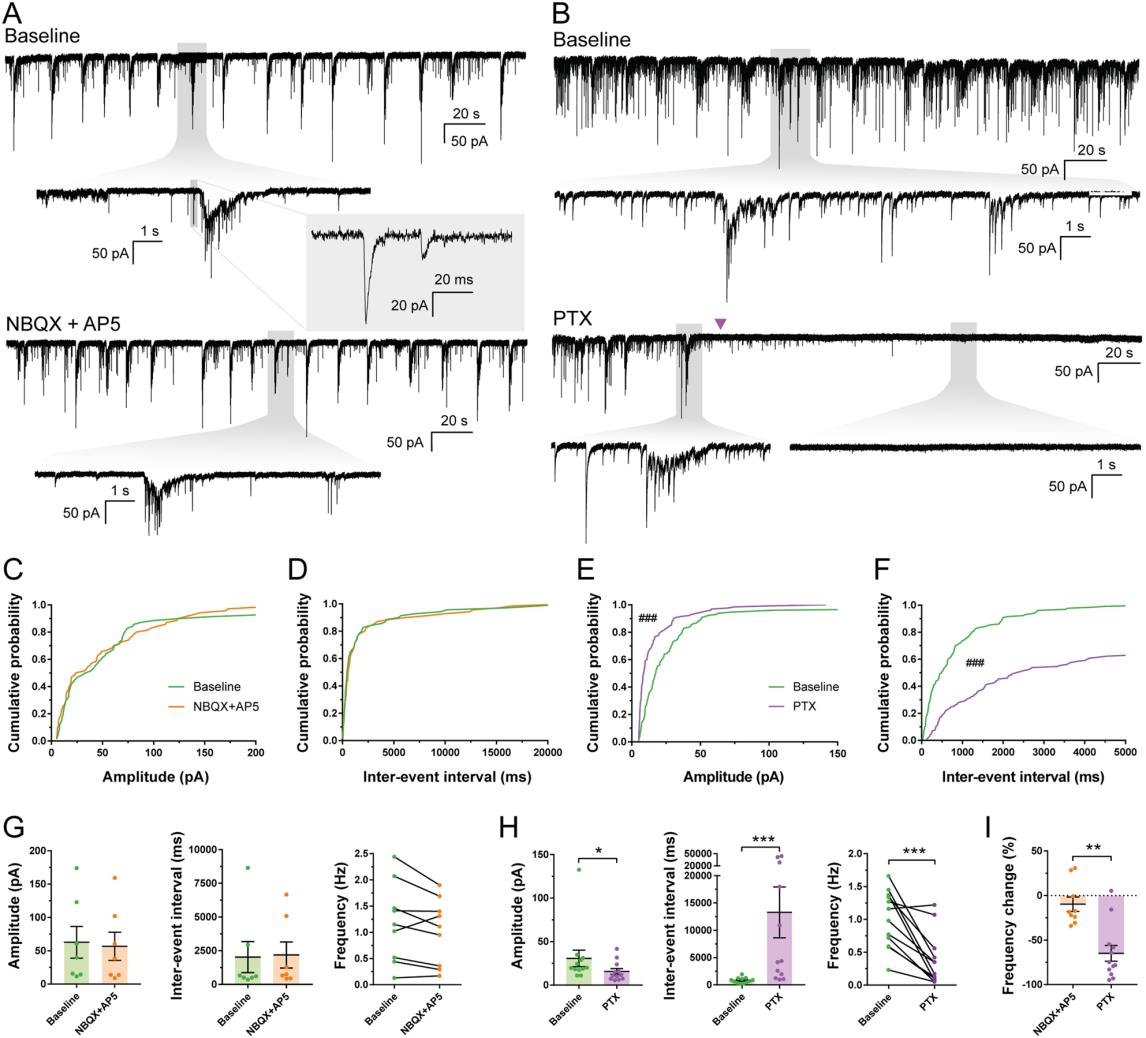
Spontaneous synaptic currents recorded in hdINs are GABAergic. hdINs exhibited spontaneous synaptic currents at 35 and 49 DIV (**A**, **B**). These spontaneous currents were reduced or abolished by the addition of PTX (1 mM, purple arrow) (**B** and **E**–**F**), but were not affected by NBQX (5 µM) and AP5 (50 µM) (**A** and **C**–**D**). Cumulative probability curves comparing the events during baseline and addition of the drugs for both amplitude (**C** for NBQX + AP5, and **E** for PTX) and inter-event interval (**D** for NBQX + AP5, and **F** for PTX). (**G**) Distribution of the mean amplitude, inter-event interval and frequency for spontaneous synaptic currents recorded from cells during baseline and after the addition of (**G**) NBQX and AP5, or (**H**) PTX. (**I**) Average frequency change after the addition of each drug in comparison to the baseline. Mean ± SEM. Kolmogorov–Smirnov test for cumulative distributions and Mann–Whitney test for comparison of means (NBQX + AP5 n = 7 and PTX n = 14; 10 events per cell and condition). Kolmogorov–Smirnov test: ^###^*p* < .0001. Mann–Whitney test: *p < .01; **p < .001; ***p < .0001; ****p < .00001.

To further explore the neuronal identity and functionality of hdINs, spontaneous synaptic currents were recorded and analyzed. These were more abundant at the latest time point, 49 DIV, indicating continuous synaptogenesis over the time points analyzed (Table S1 and Figure S2A–D). Furthermore, when blocking AMPA and NMDA glutamate receptors by applying NBQX and AP-5, respectively (Fig. 3A), no changes were observed in either amplitude (62.76 ± 11.19 pA for the baseline and 56.73 ± 10.37 pA for NBQX + AP5) (Fig. 3C and G) or inter-event interval (2027.08 ± 497.39 ms for the baseline and 2183.74 ± 514.15 ms for NBQX + AP5) (Fig. 3D and G). However, blocking GABA_A_ receptors by adding Picrotoxin (PTX) (Fig. 3B) to the artificial cerebrospinal fluid (aCSF) resulted in a significant decrease in amplitude (29.61 ± 3.46 pA for the baseline and 15.97 ± 1.67 pA for PTX) (Fig. 3E and H) and increase in the inter-event interval of post-synaptic currents (829.43 ± 86.66 ms for the baseline and 12,641.96 ± 2345.03 ms for PTX) (Fig. 3F and H). PTX increased the inter-event interval (Fig. 3F and H), reducing their frequency by 64.52 ± 8.23% on average (1.01 ± 0.12 Hz for the baseline and 0.37 ± 0.11 Hz for PTX) (Fig. 3H and I), and decreased the overall amplitude of the events, mostly by reducing the number of higher amplitude events (Fig. 3E and H). Hence, the majority of synaptic inputs were blocked by PTX but not by NBQX and AP-5 (1.18 ± 0.25 Hz for the baseline and 1.02 ± 0.21 Hz for NBQX + AP5, Fig. 3G), supporting the predominantly GABAergic nature of the synaptic network, and thereby confirming functionally the inhibitory phenotype observed with immunocytochemistry and gene expression analysis.

### Synaptic integration of hdINs with human primary cortical neurons in vitro

**Figure 4:**
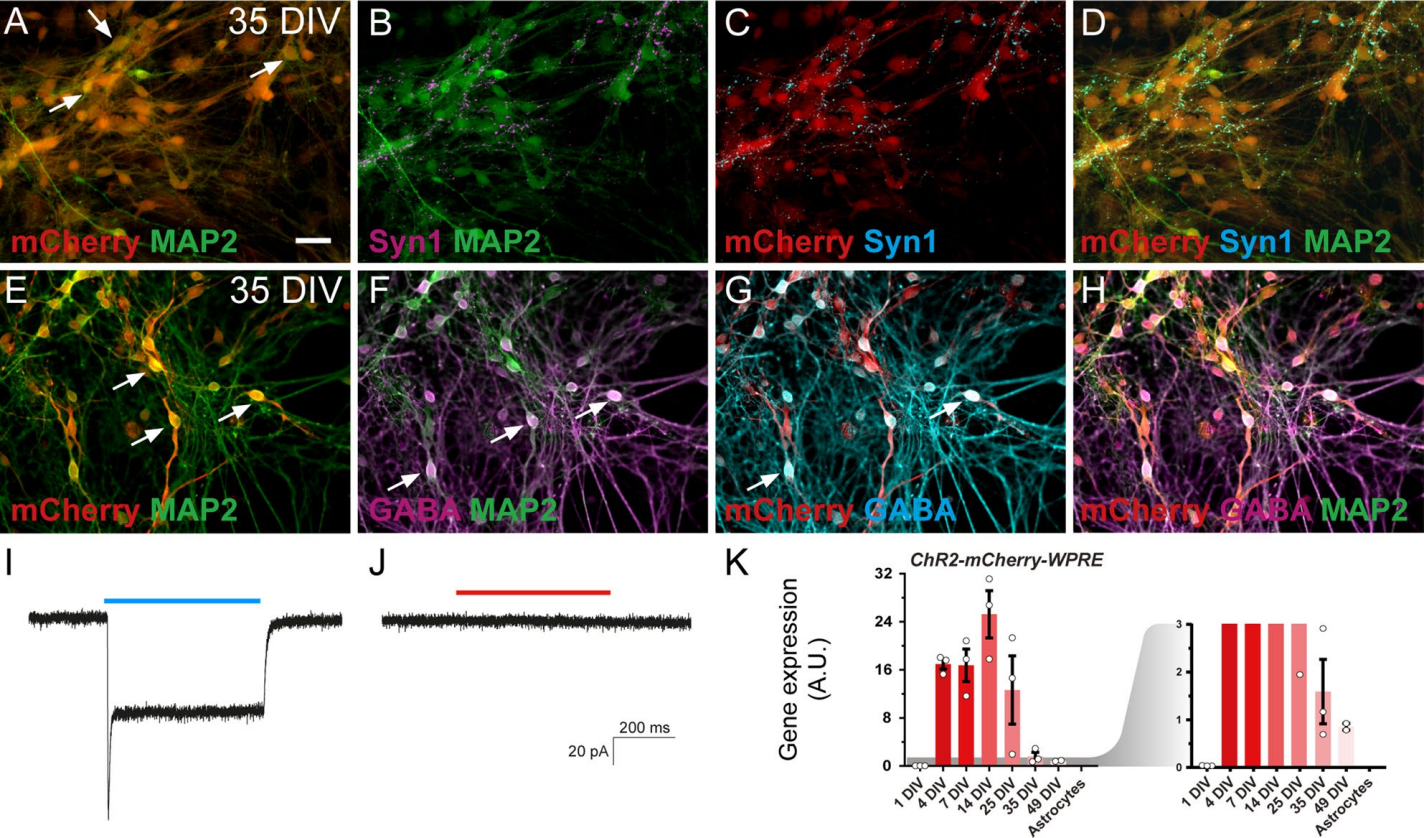
ChR2 is expressed in hdINs, enabling them to respond to blue light. (**A**–**D**) Immunocytochemistry of hdINs at 35 DIV for the neuronal marker MAP2 + in combination with the fluorescent reporter for ChR2, mCherry +, and a mature neuronal marker Syn1 + in simple differentiation cultures. (**E**–**H**) Immunocytochemistry indicating the co-expression of GABA + and mCherry + in some hdINs at 35 DIV. White arrows are examples of double positive cells for the indicated markers. ChR2-mCherry + neurons responded to blue light pulse stimulation (**I**), but not to a red-light pulse stimulation (**J**). (**K**) *ChR2-mCherry-WPRE* expression at different time points of the differentiation, shown in red. A magnified graph for *ChR2-mCherry- WPRE* values is shown in red as well on the right of the previous graph. Scale bar: 100 µm. Blue light, 460 nm. Red light, 595 nm. Blue and red line, light stimulation for the specific wavelength.

**Figure 5:**
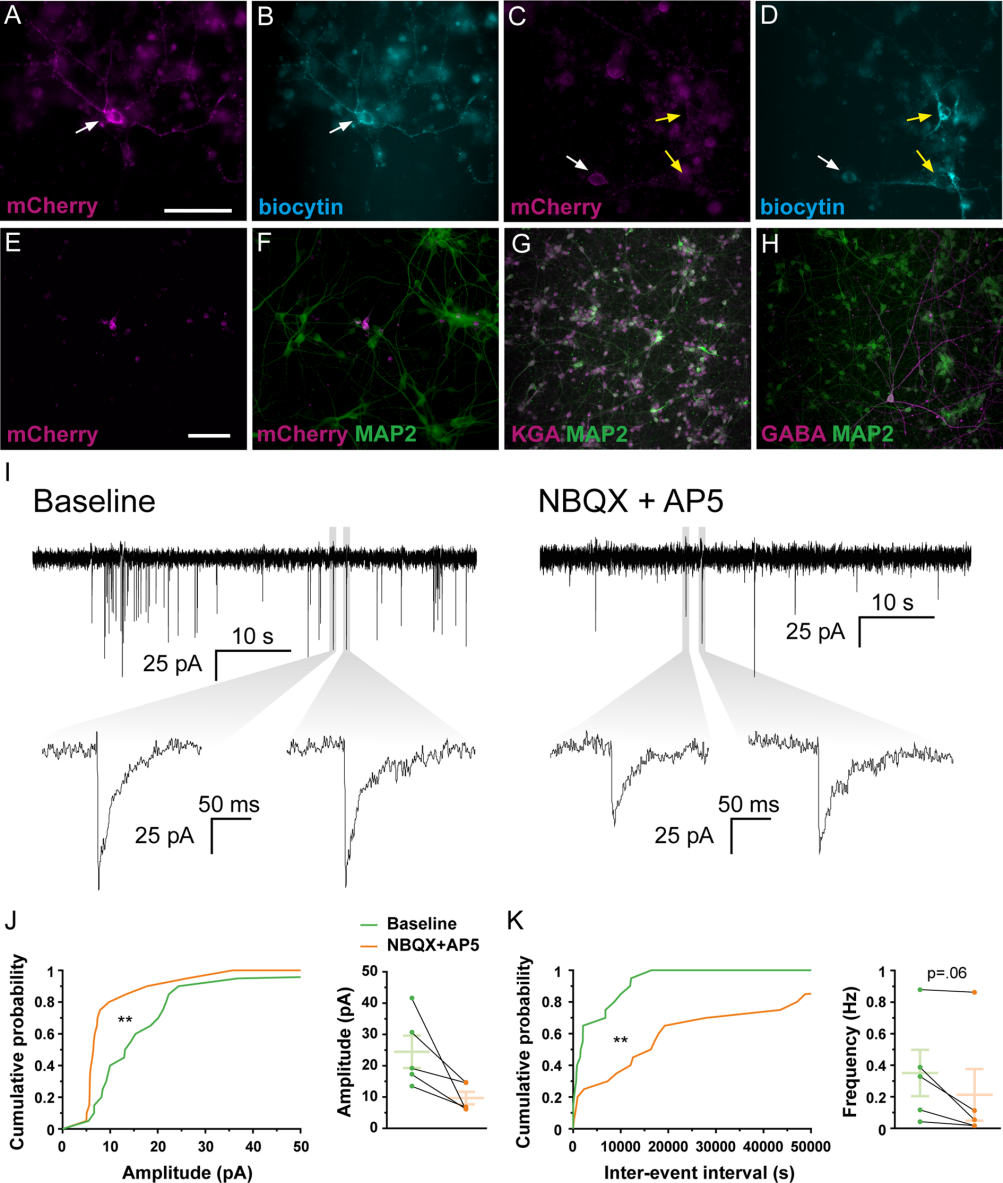
Co-cultures of human primary neurons and hdINs at 35 DIV demonstrate afferent synaptic connections from the primary neurons to the hdINs. Immunohistochemistry of co-cultures of hdINs and human primary neurons used for electrophysiological analysis at 35 DIV. Some of the recorded neurons (biocytin +, **B** and **D**) were also hdINs mCherry + (white arrows, **A** and **C**), and some were mCherry-, indicating that the latter were primary neurons (yellow arrows, **C**–**D**). (**E**, **F**) The ratio of mCherry + /MAP2 + cells to human primary neurons mCherry-/MAP2 +. (**G**, **H**) Human primary neurons were mostly glutamatergic (KGA +, **G**) and very few GABAergic (GABA +, **H**). (**I**) Spontaneous synaptic currents in whole-cell voltage-clamp mode showing afferent connections to the hdINs at 35 DIV. Some of the events disappeared when NBQX and AP5 where applied (right). (**J**, **K**) Cumulative probability curves comparing the events during baseline (green) and addition of the drugs (orange) for both amplitude (**J**, left) and inter-event interval (**K**, left). Distribution of the mean amplitude (**J**, right) and frequency (**K**, right) for spontaneous synaptic currents. Scale bar: 100 µm. Mean ± SEM. Kolmogorov–Smirnov test for cumulative distributions and Wilcoxon test for comparison of paired means (n = 5). Kolmogorov–Smirnov test: **p < .005.

**Figure 6:**
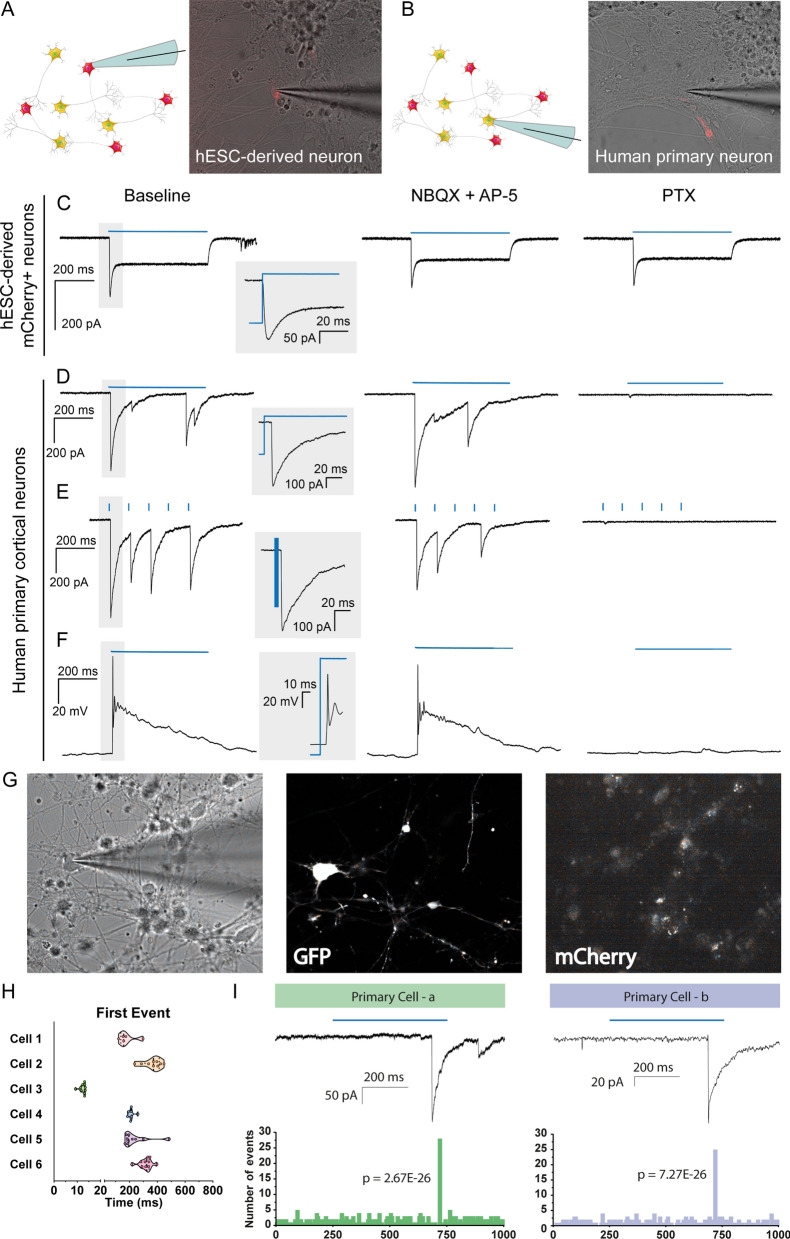
Effect of ChR2 activation on the human primary neurons co-cultured with hdINs at 35 DIV. hdINs were co-cultured at 7 DIV with primary neurons from an 8-week-old human fetus for 4 weeks. Both (**A**) hdINs, expressing mCherry as a reporter for ChR2, and (**B**) human primary neurons were recorded (n = 18 and n = 6, respectively). (**C**) The activation of ChR2 by blue light in hdINs triggered an inward current, unaffected by either NBQX + AP5 or PTX. (**D**–**F**) Light activation of ChR2 in hdINs also generated a delayed synaptic response in the surrounding primary neurons, indicating synaptic integration of those cells in the pre-existing neuronal network. Light responses were assessed during (**D**) 500 ms light pulse in voltage-clamp, (**E**) light train of 5 pulses of 3 ms with 97 ms of interval between pulses in voltage-clamp, and (**F**) 500 ms light pulse in current-clamp. These responses in the primary cells were blocked by PTX (right column, **D**–**F**), but not by NBQX + AP5 (middle column, **D**–**F**), confirming their GABAergic nature. Note that the GABAergic currents are depolarizing in the primary neurons recorded because a high chloride internal solution was used in the patch pipette. (**G**) On the left, bright-field image of a primary neuron being recorded. In the middle, same cell showing GFP + expression. On the right, the primary neuron is mCherry- (negative). (**H**) Histogram of latencies of synaptic responses for each neuron recorded. Only the first event for each trace is included in the analysis. (**I**) Two examples of primary cells displaying delayed light-induced synaptic responses. On the top, traces in voltage clamp showing the synaptic responses to blue light stimulation (blue line) and, on the bottom, histogram of latencies of increased frequency of synaptic responses for each neuron recorded. P-value for Poisson test is indicated for the highest frequency distribution time. Blue line, light stimulation. Schematics were generated and adapted using resources from Servier Medical Art^35^.

Next, we investigated whether hdINs could integrate into a human neuronal circuit in vitro and thereby modulate neuronal activity of host neurons. For this purpose, lentiviral transduction of *ChR2-mCherry* was performed before starting the differentiation of hESCs. Subsequent analyses revealed that 74.1 ± 1.35% of MAP2-positive cells also expressed mCherry (Fig. 4A–H and K), and that expressing cells were readily depolarized by exposure to ChR2-activating blue light (460 nm wavelength) similarly at 35 and 49 DIV, but not to red light (Fig. 4I–J). These results ensured that it was possible to specifically activate hdINs and study their efferent synaptic integration in human neuronal cultures. Human primary neuronal cultures were obtained from brain tissue of 8 weeks old aborted fetuses, and used as established human neuronal network after 7 DIV^15^. After four weeks in culture, human primary cortical neurons were mostly glutamatergic (Fig. 5G and I–K) with few GABAergic neurons (Fig. 5H), and displayed spontaneous synaptic bursting indicating network activity^16^. The hdINs and human primary neurons were first co-cultured for four weeks (from 7 to 35 DIV), and then whole-cell patch-clamp recordings were performed to assess the functionality and integration of hdINs in the network (Fig. 5A–D). At this time point, 35 DIV, hdINs represented 2.71% of the total number of cells within the co-culture (Fig. 5E–F) and received functional afferent connections from the human primary neurons (Fig. 5I), which had a strong glutamatergic component (Fig. 5J–K). Moreover, the activation of hdINs using blue light induced postsynaptic currents in recorded human primary neurons (Fig. 6B and D–I). For each cell, these synaptic responses occurred at a certain latency from the light stimulation onset which varied from cell to cell (Fig. 6G–I). Thus, in the recorded cells the light responses were not generated by light per se since in that case, the response would have been instantaneous without any latency period (like in the mCherry + hdIN example (Fig. 6A and C). For each recorded cell, the response latency (relative to the light pulse onset) was consistent for all repeated stimulations (Fig. 6H–I) confirming stable functional synaptic connections onto human primary cortical neurons after 4 weeks of co-culture. Moreover, the delayed synaptic response was blocked by PTX (Fig. 6D–F, right column), but not by NBQX and AP-5 (Fig. 6D–F, middle column), demonstrating the GABAergic nature of the synaptic connections originating from the light response of transplanted cells, and confirming to the morphological evidence that hdINs were indeed of GABAergic phenotype even when co-cultured with human primary neurons.

### Survival, differentiation, and synaptic integration of hdINs transplanted into human tissue from brain resections of drug-resistant epileptic patients

**Figure 7:**
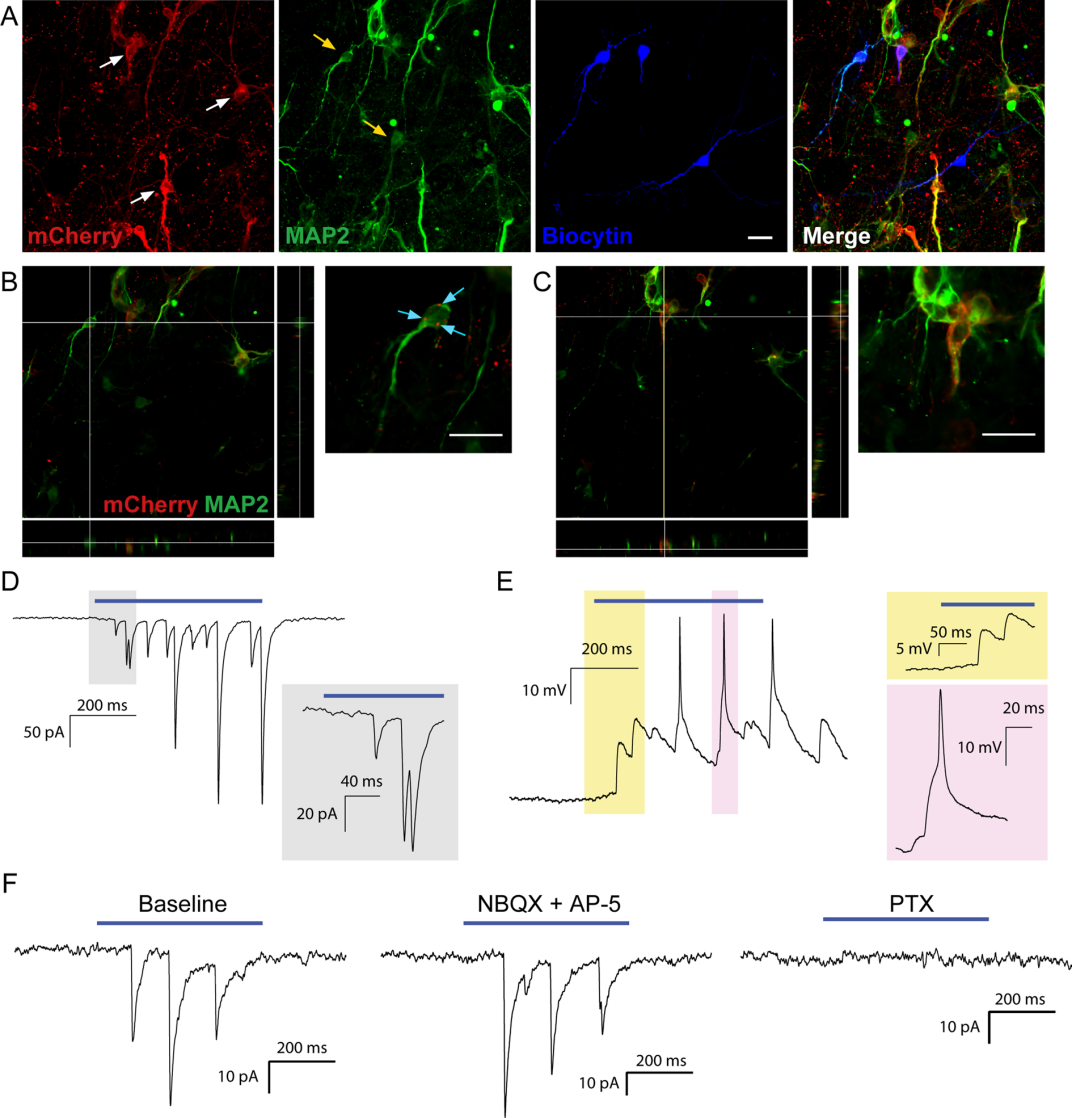
Grafted hdINs established functional efferent synaptic connections to the host adult human neurons. (**A**) hdINs survive and differentiate after transplantation onto human organotypic brain cultures for 4–6 weeks AT. hdIN-mCherry + neurons are indicated with white arrows, and host neurons are marked with yellow arrows (mCherry-/MAP2 +). (**B**) Orthogonal projection of a host neuron and magnification on the right. There are mCherry + processes surrounding the host neuron (cyan arrows). (**C**) Orthogonal projection of a hdIN mCherry + neuron, which is also MAP2 +. (**D**) Delayed synaptic response in voltage-clamp mode of the host neuron after optogenetic stimulation of the hdINs by a 500 ms light pulse, indicating synaptic integration of those cells in the pre-existing neuronal network at 4 weeks AT. (**E**) Delayed synaptic response in current-clamp mode of the host neuron after optogenetic stimulation of the hdINs, illustrating changes of the membrane potential (magnification in yellow), that eventually leads to the generation of an AP (magnification in pink). Note that the GABA_A_ receptor-generated currents are depolarizing in the host recorded cell because a high chloride solution was used in the patch pipette. These responses in the host neurons were blocked by PTX (right column in **F**), but not by NBQX + AP5 (middle column in **F**), confirming their GABAergic nature. Scale bar: 20 µm.

To determine if survival and synaptic integration of dhINs was possible in chronic epileptic tissue, we grafted hdINs onto cultured slices obtained from patients undergoing surgery for drug-resistant epilepsy. The hdINs were transplanted after 7 DIV onto organotypic cultures of resected adult brain from one temporal lobe epilepsy patient (cortical slices, n = 4 and hippocampal slices, n = 3) and one focal cortical dysplasia patient (cortical slices, n = 3), and kept in culture for 4 to 6 weeks. The grafted hdINs expressed the neuronal marker MAP2 + (Fig. 7A and C) and exhibited extensive arborizations (Fig. 7A) after 4 weeks in culture. hdINs were functional and showed intrinsic properties comparable to mature neurons at both 4 and 6 weeks after ex vivo transplantation (Figure S3 A–D). Moreover, hdINs responded to blue light with photocurrents (Figure S3 E–G) and received afferent synaptic inputs from the neighboring cells (Figure S3 H–I, n = 28 cells). In some of the hdINs, delayed inward synaptic currents were observed during the 500 ms light pulse (after the direct light response, Figure S3 J, red arrow), presumably generated by afferent synaptic connections from other hdINs (Figure S3 J, green arrows). Optogenetic activation of hdINs induced postsynaptic currents in host neurons already at 4 weeks AT (Fig. 7A–B and D–E, n = 7), which were blocked by PTX but not NBQX + AP5 (Fig. 7F; n = 4), as observed in the co-cultures with human primary neurons. These experiments demonstrate that hdINs survive and differentiate into GABAergic phenotype even when transplanted into epileptic human brain tissue.

Taken together, our findings demonstrate the capacity of the hdINs to differentiate into mature functional neurons, integrate into a human neuronal network by receiving functional afferent connections and, most importantly, by forming efferent synaptic connections with neighboring human host neurons enabling precise spatiotemporal modulation of the human neuronal network, including epileptic tissue.

## Discussion

Here we demonstrate, for the first time to our knowledge, that hESC-derived GABAergic neurons can form functional *efferent* synaptic connections onto human primary neurons in vitro, and to host neurons in human epileptic tissue after transplantation.

By validating and adapting the differentiation protocol published by Yang, et al*.* (2017)^14^ to overexpress only two transcription factors critical for the GABAergic fate (*Ascl1* and *Dlx2*), we obtained cultures with high yield of GABAergic neurons. This protocol also required a shorter time of differentiation compared to those published elsewhere based on the use of small molecules^9–11,17^. Human ESCs differentiated to functional neurons, exhibiting a fast TTX-sensitive sodium current and a sustained TEA-sensitive potassium current, allowing cells to fire action potentials already at 25 DIV. Efferent synaptogenesis was apparent at 35 DIV and proved to be predominantly GABAergic since it was blocked by PTX, but not affected by NBQX and AP-5.

One of the potential future applications of these cells is cell-based replacement therapy. As mentioned before, GABA-releasing interneurons are responsible for the modulation of neuronal network activity in the brain, and therefore their alteration or absence disrupts the excitatory-inhibitory balance in the neuronal circuits leading to neurological disorders^8^. The substitution and/or replacement of those aberrant or missing interneurons could become a potential therapeutic approach for disorders such as schizophrenia, autism, and epilepsy. A translational development of this approach towards the clinical applications will require homogeneity and reproducibility of the cell differentiation. In this regard, the present study achieved high yield of GABAergic interneurons of two major subtypes expressing CB and CR, respectively, as well as forebrain markers such as FOXG1^14^. The markers of striatal GABAergic projection neurons CTIP2 and DARPP-32 were absent, suggesting exclusive generation of forebrain GABAergic interneurons.

Another important aspect is the validation of animal data in human-derived tissue to ensure that outcomes are not specific just to the rodent brain. Currently, however, most of the translational research is being focused on animal models. This may have contributed to the failure of some therapies when tested in human clinical trials. It is believed that for cell replacement therapy, a functional integration of transplanted neurons into the existing brain circuitry is needed. Previous studies have used optogenetics for addressing this issue^18–21^. For example, Cunningham, et al*.* (2014) transplanted human stem cell-derived GABAergic interneurons expressing ChR2 into the hippocampus of a pilocarpine mouse model of epilepsy, and suppressed seizures and behavioral abnormalities either by spontaneous firing or by optogenetic stimulation^22^. Weick, et al*.* (2011) also used optogenetics to demonstrate functional synaptic integration in vitro of hESC-derived excitatory neurons into a pre-existing mouse neuronal circuit^16^. Using an optogenetic approach, we demonstrate that in cultured human primary cortical neurons and in an adult epileptic human neural circuitry, grafted hESC-derived GABAergic neurons integrate, forming functional efferent synapses. The human cell co-culture paradigm and the ex vivo transplantation onto human epileptic organotypic cultures shown here for testing the efferent synaptic integration of derived neurons could be considered as a useful platform incorporated into the roadmap of clinical translation.

One interesting aspect demonstrating efferent synaptic integration of the derived GABAergic neurons is the response pattern recorded in co-cultured human primary neurons (Fig. 6D–F). Light responses occurred with a consistent latent period upon light stimulation (Fig. 6G–I), that differed from cell to cell. The consistent latent period for each cell indicates that the effect is light dependent, rather than spontaneous (Fig. 6G–I). The variability between cells could be due to multiple causes: (i) Varied length of a polysynaptic chain between the hdINs and recorded primary neurons. This is enabled by GABA being an excitatory neurotransmitter at this stage of neuronal development, due to higher intracellular concentration of chloride, which depolarizes the membrane upon GABA_A_ receptor activation^23–29^. In support, Dzhala et al*.* (2005) demonstrated that the peak of *SLC12A2* expression (encoding NKCC1) in human brain occurs at 35 postconceptional weeks (PCW), decreasing rapidly during the first year of life (54–92 PCW). It is in early childhood (92–210 PCW, approximately 1–3.3 years) when *SLC12A5* expression levels (encoding for KCC2 protein) takes over^30^. (ii) The differential expression of ChR2 in the hdINs that leads to variability in timing to the first action potential. (iii) Variability of neurotransmitter release from grafted cells by direct depolarization of presynaptic terminals by light.

Various types of derived neurons engrafted in human neuronal cultures receiving functional afferent synaptic connections, as also shown here, have been reported previously^31,32^. However, evidence for functional efferent connections from the converted neurons to the human fetal primary cortical neurons or the organotypic human brain slices has been lacking. Our study provides the first evidence to our knowledge that hESC-derived neurons are capable of forming functional efferent synaptic connections to human neurons, and thereby possess the potential to modulate activity and network excitability of the human neuronal network.

## Methods

### Stem cell maintenance

H1 (WA01) ESC were obtained from WiCell Research Resources (Wicell, WI). Human ESC were maintained as feeder-free cells on Matrigel-coated (Corning) plates using Essential 8 Flex medium (E8F; Gibco) and passaged as colonies using ReLeSR (Stem Cell Technologies).

### Mouse primary glial cell culture

All animals were bred at the local animal facility and kept in 12 h light/dark cycle with access to food and water ad libitum. All procedures were approved by the Malmö/Lund Animal Research Ethics Board, ethical permit number 02998/2020.

Mouse primary glial cells were harvested from the cerebral cortex of newborn C57Bl6/J mice at P3 to P5. Briefly, mice pups were separated from the mum and decapitated without anesthesia using scissors. Thus, the brain was extracted and dissected, and the cerebral cortex was cut, homogenized, and digested with trypsin for 30 min at 37 °C. Cells were dissociated mechanically, passed through a cell strainer, and plated onto T75 flasks coated with poly-D-lysine (PDL; Sigma-Aldrich) in MEM (Gibco) supplemented with 5% fetal bovine serum (FBS; Sigma), 0.4% D-Glucose (w/v; Sigma), 2% B-27 (Gibco), 1% GlutaMAX (Gibco) and 1% Penicillin–Streptomycin. Primary glial cells were maintained and passaged at confluency using trypsin until a maximum of 5 passages, being passaged at least once before being used for co-culture with the hdINs.

### Lentiviral constructs and virus generation

High-titer of third-generation lentiviral particles was produced using PEI for transfection of the 293 T cells in biosafety level 2 environment^33^. Lentiviral particles were obtained for the following constructs: hSyn1-ChR2(H134R)-mCherry-WPRE (obtained by cloning at the lab, from Addgene #20945), and the TetOn system consisting on FUW-rtTA (Addgene #20342), FUW-TetO-Ascl1-T2A-puromycin (Addgene #97329) and FUW-TetO-Dlx2-IRES-hygromycin (Addgene #97330).

### Differentiation of hESC-derived neurons

Before starting the differentiation procedure, hESC were transduced with lentiviral particles carrying hSyn1-ChR2(H134R)-mCherry-WPRE, and the TetOn system rtTA/Ascl1-puro/Dlx2-hygro at MOI 5/2.5/2.5 respectively; in fresh E8F medium containing 10 µM ROCK inhibitor Y-27632 (Y; Stem Cell Technologies).

hdINs were generated as described in Yang *et al.* (2017)^14^, with the addition of some modifications. Human ESC were passaged as single cells using Accutase (Stem Cell Technologies). Cells were plated in six-well plates coated with Matrigel at a density of 3 × 10^5^ cells/well in E8F containing Y on –1 DIV. At 1 DIV, the cultured medium was replaced with N2 medium consisting of DMEM/F12 (Gibco) supplemented with N2 Supplement (1:100; Gibco) and containing doxycycline (DOX; 2 g/l; Sigma-Aldrich) to induce the TetO gene expression. DOX was added to the media for 14 days. At 2 DIV, an antibiotic-resistance selection period was started by adding puromycin (puro; 0.5 µg/ml; Gibco) and hygromycin (hygro; 750 µg/ml; Invitrogen) to the fresh media. At 5 DIV, the selection period ended and cells were cultured in N2 medium containing DOX and cytosine β-D-arabinofuranoside (Ara-C; 4 µM, Sigma). After a week in culture, at 7 DIV, cells were detached into a single-cell suspension using Accutase and plated together with mouse primary glial cells on Matrigel-coated glass coverslips in a 24-well plate (3–5 × 10^5^ and 5 × 10^4^ cells/well respectively). At this point, the medium was replaced with Growth medium consisting of Neurobasal medium supplemented with 2% B27, 1% GlutaMAX and 5% FBS. From 7 DIV until the day of the analysis (25 DIV, 35 DIV, and 49 DIV), half of the medium was replaced for fresh one every 2–3 days. Additionally, from approximately 10 DIV onwards Ara-C was added to the medium to inhibit glial cell proliferation, and from 15 DIV until the last time point BDNF (14 ng/ml, R&D Systems) was also added. Importantly, DOX was withdrawn from the medium at 14 DIV.

### Derivation of human fetal primary cortical cells and co-culture with hdINs

Human primary cortical cells were derived from the cerebral cortex of aborted human fetuses (8 weeks of age) according to guidelines approved by the Lund-Malmö Ethical Committee (Ethical permit number: Dnr 6.1.8-2887/2017) as described in Miskinyte et al*.* (2017)^31^. The tissue was carefully dissected, minced into small pieces, and then triturated with a pipette tip into a single-cell suspension. The cells were washed with Neurobasal (Gibco)-based medium supplemented with B27, and plated onto poly-d-lysine (Sigma-Aldrich)/fibronectin (Life Technologies) (both 10 μg/mL)-coated glass coverslips at a density of 50,000 cells/well and maintained in the same medium until co-culturing. For a subset of experiments, human primary neurons were transduced with lentiviral vectors carrying EF1α-GFP prior to the co-culture (Fig. 6G).

hdIN precursors were detached at day 7 of differentiation and seeded onto human primary cortical cells at a density of 15 × 10^4^ cells/well. Then, both cell types were cultured together following the differentiation protocol described above for 4 weeks (reaching 35 DIV for hdINs). Due to the proliferative nature of the neuronal precursors from the human fetuses, at 35 DIV hdINs represented a 2.71% of the total number of cells in the culture, calculated by counting 2.01 ± 0.27% mCherry + cells which are the 74.1 ± 1.35% of the total hdINs. So, probability of recording from hdIN mCherry- cells instead of human primary neurons was less than 1%. Those values are in coherence with the number of cells we would expect from previous counting at 35 DIV in a regular differentiation with 500.000 seeding cells at 7 DIV. The number of cells were 61.5 ± 5.42 cells in an area of 680 × 510 µm, and in the co-culture scenario where the seeding cells at 7 DIV were 15 × 10^4^ cells/well (3.3 times less) the number was 7.83 for the same area. Hence, the co-culture environment does not affect the survival of the differentiated cells.

### Organotypic cultures of adult human brain tissue and transplantation of the hdINs

Resected neocortical and hippocampal tissue were obtained from patients undergoing surgical treatment for drug-resistant epilepsy (n = 2). The use of resected patient tissue and following procedures were approved by the local Ethical Committee in Lund (#212/2007) and were performed in accordance with the Declaration of Helsinki. Written informed consent was obtained from all subjects prior to each surgery. Patient information:

Patient 1: TLE resection, male, age 49 years, duration of epilepsy 4 years; approximately 3 seizures/week; medication at time of surgery: lamotrigine; pathology: signs of previous limbic encephalitis.

Patient 2: FCD resection, female, age 27 years, duration of epilepsy 16 years; approximately 3–5 seizures/month; medication at time of surgery: lamotrigine and Trileptal®; pathology: FCD type IIId.

The tissue slices were derived and handled as previously described^34^. Briefly, tissue was transported from the surgery room to the electrophysiology laboratory in an ice-cold sucrose-based slushed cutting solution, containing in mM: 200 sucrose, 21 NaHCO_3_, 10 glucose, 3 KCl, 1.25 NaH_2_PO_4_, 1.6 CaCl_2_, 2 MgCl_2_, 2 MgSO_4_ (all from Sigma-Aldrich, Sweden), adjusted to 300–310 mOsm, 7.4 pH. At the laboratory, the tissue was then transferred into the same type of solution, continuously bubbled with 95% O_2_ and 5% CO_2_. The 300 µm slices were cut with a vibratome (Leica VT1200S) and transferred to a rinsing media, containing: HBSS (Life Technologies), HEPES (4.76 mg/ml; Sigma), Glucose (2 mg/ml; Sigma), Penicillin/Streptomycin solution (50 ul/ml; Life Technologies). After 15 min in the rinsing media, slices were transferred to membrane inserts (Millipore, PIHP03050) in six well plates filled with slice culture medium: BrainPhys medium (Stemcell Technologies) supplemented with B27, Glutamax (1:200), Penicillin/Streptomycin solution (10 ul/ml; Life Technologies), and incubated in 5% CO_2_ at 37 °C. The organotypic slices were kept in culture for at least 1 day before hdINs were detached at 7 DIV and seeded onto the tissue. Organotypic cultures were kept for 30 min in the incubator after seeding the cells in an air-liquid interface, then media was added on top to cover the surface.

### Immunocytochemistry

Both hdINs and human primary neurons, plated on glass coverslips were rinsed with phosphate-buffered saline (PBS), fixed in 4% paraformaldehyde (PFA) for 20 min at room temperature (RT), and washed three times in KPBS. For GABA detection, coverslips were fixed with 0.25% glutaraldehyde in 4% PFA instead. Then, coverslips were pre-incubated in blocking solution for 1 h (10% normal serum and 0.25% Triton X-100 in KPBS). Primary antibodies diluted in the blocking solution were incubated overnight at 4 °C (Table S2). Coverslips were washed three times in KPBS and further incubated with Alexa Fluor 488, 555 and 647 conjugated donkey or goat secondary antibodies (1:1000, Jackson Immunoresearch, PA) against the respective primary antibodies, diluted in blocking solution for 1.5 h at RT. Nuclei were counterstained with Hoechst 33342 (1:1000) diluted in the last rinsing with PBS before mounting with Dabco mounting media. Images were acquired by an epifluorescence microscope (Olympus BX61).

For staining human organotypic cultures, slices were fixed overnight at 4 °C with 4% PFA and changed to KPBS after. Then, slices were incubated for 1 h at RT in permeabilization solution (0.02% BSA + 1% Triton X‐100 in PBS) and 2 h at RT in blocking solution (5% normal serum + 1% BSA + 0.2% Triton X‐100 in PBS). Primary antibodies were diluted in blocking solution and incubated for 48 h at 4 °C. Then, slices were incubated again in blocking solution 2 h at RT. Secondary antibodies were applied in blocking solution for 48 h at 4 °C. Finally, nuclei were stained with Hoechst for 20 min at RT before sections were mounted. Images were acquired by confocal microscopy (Nikon Confocal A1RHD microscope).

### Gene expression analysis

RNA was extracted from cells using RNeasy mini kit (Qiagen) and then reversed to cDNA using Maxima First Strand cDNA Synthesis Kit for RT-qPCR (Thermo Fisher Scientific). For quantitative PCR, cDNA was prepared with PowerUp SYBR Green Master Mix (Thermo Fisher). Candidate genes related to different stages of neurodevelopment and neuronal subtypes were selected for gene expression analysis. A complete list of the primers used is shown in Table S3. Three different biological replicates from different batches of differentiation were used for each time point. All the samples were run in technical triplicates, and the average Ct-values were used for calculations. Data was represented using the ΔCt method, in which all gene expression values are calculated as the average change based on two different housekeeping genes (*ACTB* and *GAPDH*).

### Electrophysiology

Human dIN precursors were grown on coverslips from day 7 of differentiation when they were co-cultured with mouse primary glial cells. For in vitro recordings, the coverslips were transferred to the recording chamber containing aCSF (in mM): 129 NaCl, 21 NaHCO_3_, 10 glucose, 3 KCl, 1.25 NaH_2_PO_4_, 2 MgSO_4_, and 1.6 CaCl_2_, adjusted to 300–310 mOsm, pH 7.4, heated to 32 °C and continuously bubbled with carbogen (95% O_2_ and 5% CO_2_).

Target cells were identified under fluorescent light (520 nm) for mCherry^+^ and all the recorded cells were visualized for whole-cell patch-clamp recordings using infrared differential interference contrast video microscopy (BX51WI; Olympus). The glass capillary patch pipette (tip resistance between 2.5 and 6 MΩ) was backfilled with a solution containing in mM: 122.5 K-gluconate, 17.5 KCl, 10 KOH-HEPES, 0.2 KOH-EGTA, 2 Mg-ATP, 0.3 Na_3_GTP, and 8 NaCl, pH 7.2–7.4 (mOsm 290–300; all from Sigma-Aldrich). Moreover, biocytin (0.5–1 mg/ml, Biotium) was dissolved in the pipette solution for *post-hoc* identification of recorded cells. All recordings were performed using an EPC10 double patch-clamp amplifier (HEKA Elektronik, Germany), sampled at 10 kHz with a 3 kHz Bessel anti-aliasing filter and using PatchMaster software for data acquisition.

After the formation of a GΩ seal, the patch was ruptured giving direct access to the intracellular compartment. Resting membrane potential (RMP) was determined in current-clamp mode at 0 pA immediately after establishing the whole-cell configuration. Series resistance (Rs) and input resistance (Ri) were calculated from a 5 mV voltage pulse applied through the patch pipette and monitored throughout the experiment. A series of square current steps of 500 ms duration from − 40 to 200 pA in 10 pA steps, were applied at a membrane potential of approximately − 70 mV with holding current as needed, to determine the cells' ability to generate action potentials (AP). Sodium and potassium currents were evoked by a series of 100 ms long voltage steps ranging from − 90 to + 40 mV in 10 mV steps and their sensitivity to 1 µM TTX and 10 mM TEA was determined. AP characteristics were assessed by administration of a depolarizing ramp current over 1 s, from a holding potential of − 70 mV, starting with a 0–25 pA ramp and up to a 0–300 pA ramp in various cells. Spontaneous postsynaptic currents were recorded at − 70 mV.

#### Drugs and concentrations

 Drugs and concentrations: All the used drugs were applied in an aCSF solution perfusing the recording chamber, with the following concentrations: *N*-Methyl-d-aspartic acid (NMDA) receptor blocker (2R)-amino-5-phosphonovaleric acid (D-AP5) 50 µM (Abcam); a-amino-3-hydroxy-5-methyl-4-isoxa-zolepropionic (AMPA) receptor blocker 2,3-dihydroxy-6-nitro-7-sulfamoyl-benzo[f]quinoxaline-2,3-dione (NBQX) 5 µM (Abcam Biochemicals); GABA_A_-receptor blocker picrotoxin (PTX) 100 µM (Tocris); Tetrodotoxin (TTX) 1 µM (Abcam); and tetraethylammonium (TEA) 10 mM (Abcam).

#### Optogentics

For the optogenetic activation of ChR2-expressing cells, blue light of 460 nm wavelength was applied with a LED light source (Prizmatix, Modiin Ilite, Israel) connected to the microscope via a waveguide, illuminating the slice through the water immersion 40 × microscope objective. Red light (595 nm) was applied as a negative control. The frequency and duration of light pulses were programmed and controlled within the Patchmaster software. Stimulation of ChR2-expressing cells was done either by continuous application of the blue light for 500 ms (pulse) or 5 pulses of 3 ms separated by 97 ms intervals (train).

#### Human primary neuron and host neurons from the human tissue recordings

Human primary neurons and host neurons from the human organotypic cultures were identified by infrared differential interference contrast microscopy, not expressing mCherry under fluorescent light. For a subset of experiments, these cells were identified by GFP + expression (Fig. 6G). The patch pipette was backfilled with a solution containing in mM: 140 KCl, 10 HEPES, 0.2 EGTA, 4 Mg-ATP, 0.4 Na_3_GTP, and 10 NaCl, pH 7.2–7.4 (mOsm 290–300; all from Sigma-Aldrich).

### Statistical analysis

Quantification of the number of immunoreactive cells was performed in five randomly selected 20 × visual fields for each coverslip from at least three independent cell differentiations. Results for the different markers were expressed as a percentage of the total number of MAP2 + cells. The number of MAP2 + cells varied between 26 to 130 cells in each area counted, with a mean of 52.25 ± 1.78 cells/area, and a minimum of 600 cells were counted for each marker and time point.

Whole-cell patch-clamp recordings were analyzed offline with Igor Pro (Wavemetrics) and Python. AP amplitude was measured on ramp recordings from threshold to peak and AP duration was measured as the width at the threshold. The amplitude of the afterhyperpolarization (AHP) was measured on depolarizing square current steps as the difference between the AHP peak and the AP threshold. Spontaneous postsynaptic currents were detected and analyzed using a custom Python script (https://github.com/AMikroulis/xPSC-detection). Voltage-clamp recordings were low-pass filtered at 400 Hz. An averaged postsynaptic current template generated from hdINs recordings was used for the detection (Figure S2E). Events with a correlation coefficient to the template of 0.6 or greater were included in the analysis (Figure S2F). The rise and decay times were measured as the interval between 20 and 80% of the maximum amplitude. Before the statistical analysis, four exclusion criteria were applied: (1) events with < 5 pA of amplitude were excluded (due to the amplifier’s intrinsic noise floor at 4 pA p-p); (2) events with rise-time > 3 ms were excluded; (3) events with decay-time > 20 ms were excluded; and (4) events with decay-time shorter than 1.5 times the rise-time of the event were also excluded. For an equal statistical representation of the different neurons analyzed, an equal number of events were analyzed for all neurons.

Statistical analysis of the data was performed using Prism 7 (GraphPad). The Mann–Whitney test was used for comparison of medians, one-way ANOVA with Tukey’s post hoc test for multiple comparisons of means and Wilcoxon test for comparison of paired data. Fisher’s exact test was used for comparison of proportions. The level of significance for the tests was set at *p* < 0.05. The Kolmogorov–Smirnov test was used for distribution comparisons of spontaneous currents and the significance was set to *p* < 0.01. All data is presented in the figures as Mean ± SEM. Outlier detection test was applied for the analysis of spontaneous synaptic activity in Fig. 3 and Figure S2, detecting only one outlier that was discarded from the analysis although it did not affect the statistical significance.

## Supplementary Information

Supplementary Information.
