# Supplementary material for: Human stem cell-derived GABAergic neurons functionally integrate into human neuronal networks
Source: Sci Rep. 2021 Nov 11;11:22050. doi: 10.1038/s41598-021-01270-x (PMC8585944; doi:10.1038/s41598-021-01270-x)
Supplement: Supplementary file 1 — Supplementary Information. [file 41598_2021_1270_MOESM1_ESM.pdf]

Supplemental items

Figure S1

Gene expression profile during the cell differentiation. RT-qPCR analysis for the gene expression of oligodendrocyte precursors marker (*PDGFRα*, blue), the MGE marker (*NKX2.1*, orange), and the main interneuron subtypes markers (pink) together with *PPP1R1B* gene (pink), which is expressed in the striatal GABAergic projecting neurons known as medial spiny neurons. Mean ± SEM (n = 3 differentiation batches for condition / time point).

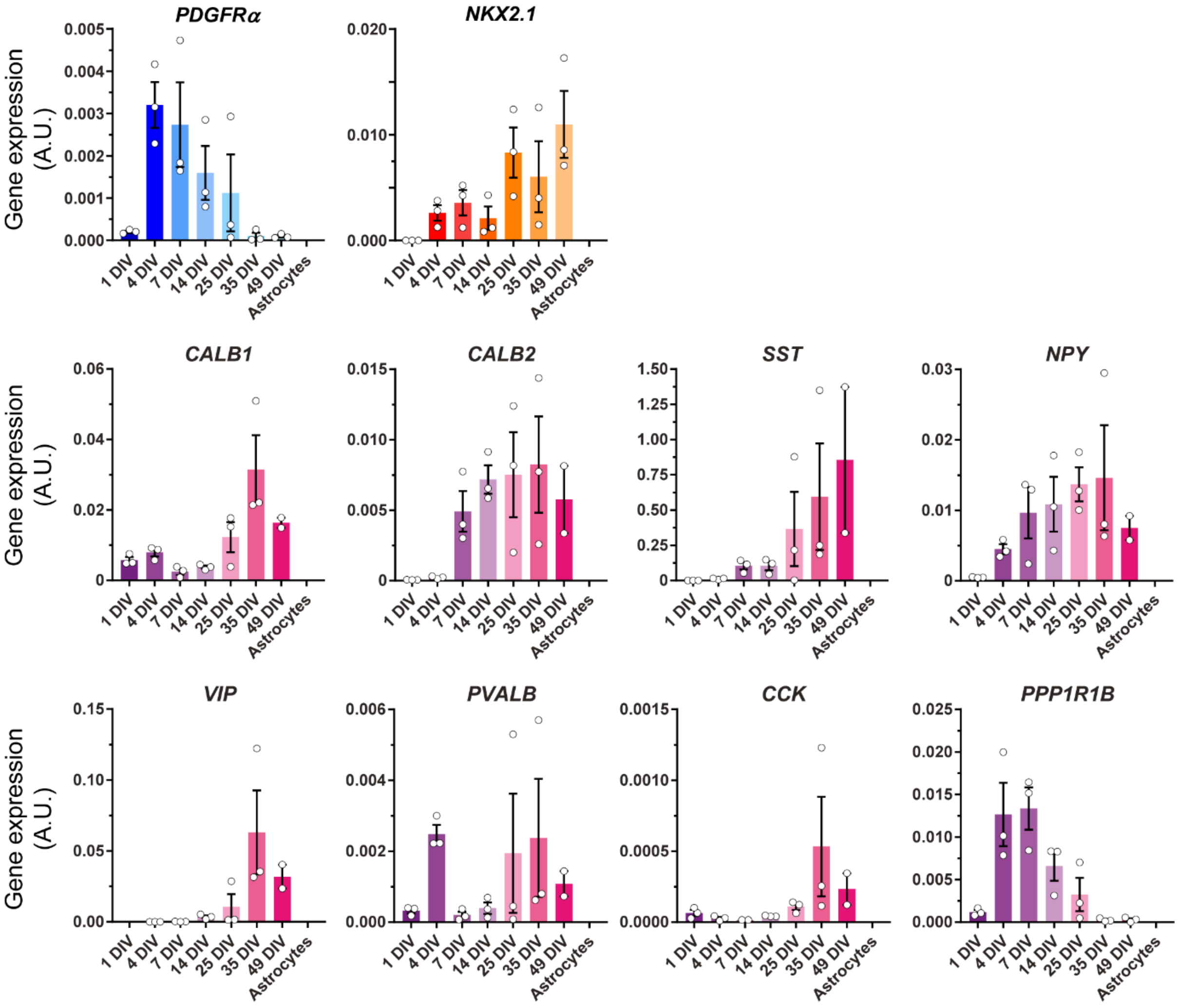

Figure S2

Spontaneous synaptic currents recorded in hdINs at 35 and 49 DIV. hdINs exhibited spontaneous synaptic currents at 35 and 49 DIV. Approximately 80% of the events at 49 DIV had larger amplitudes than at 35 DIV (A). This was accompanied by a higher number of events at 49 DIV (C) compared to 35 DIV. Nevertheless, distribution of the means for both amplitude (B) and inter-event interval (D) were similar between 35 and 49 DIV. (E) Template used for detecting events. (F) Example of event detection using a 0.6 correlation coefficient to the model (E), before further filtering using exclusion criteria. Above in black there is the recording in voltage-clamp and below in red is the automated event detection indicated with red arrows. Mean ± SEM. Kolmogorov-Smirnov test for cumulative distributions and Mann-Whitney test for comparison of means (35 DIV n=13 and 49 DIV n=8, 10 events per cell and condition). ##, p<.001.

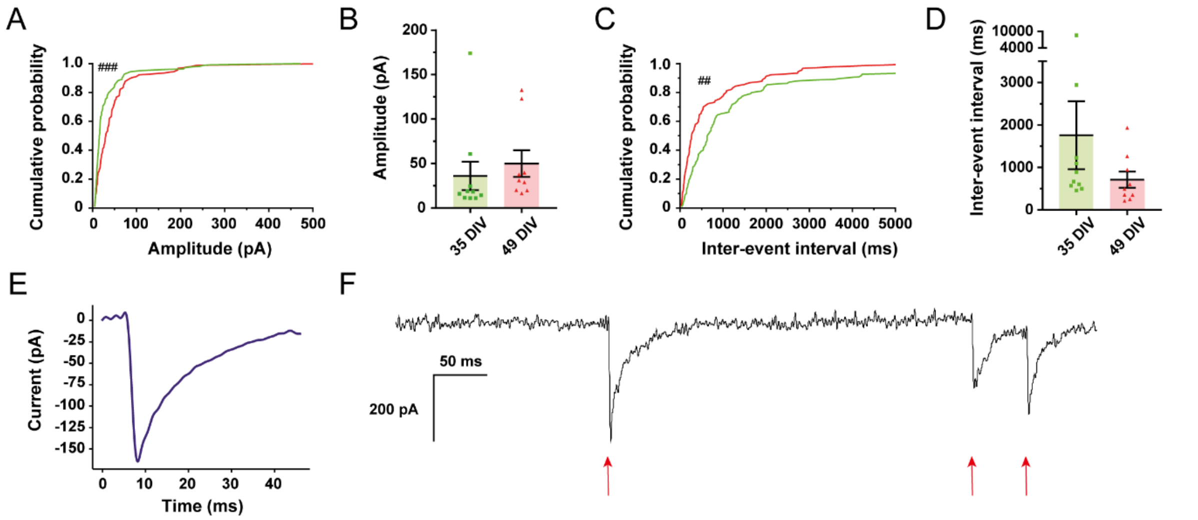

Figure S3

Electrophysiological properties and spontaneous synaptic currents in dhINs 4-6 weeks after *ex vivo* transplantation onto adult human organotypic brain cultures. Grafted hdINs were able to respond with multiple action potentials to depolarizing current pulses (A) and ramps of depolarizing current (B), comparable to mature neurons. Magnification of a current induced action potential in green. (C) Expanded current traces illustrating the sodium current and (D) the potassium current activated during voltage pulses ranging from -90 mV to +40 mV in 10 mV steps. (E-G) Optogenetic stimulation of ChR2 in hdINs triggered inward currents. Light responses were assessed during (E) 500 ms light pulse in voltage-clamp, (F) light train of 5 pulses of 3 ms with 97 ms of interval between pulses in voltage-clamp, and (G) 500 ms light pulse in current-clamp. (H, I) Spontaneous synaptic currents in whole-cell voltage-clamp (H) and current-clamp (I) mode showing afferent synaptic activity in the hdINs. (J) Optogenetic stimulation of ChR2 in hdINs triggered immediate inward currents (red arrow) and, in some cells, also delayed light responses (green arrow) later in the light pulse generated most likely by another hdINs connecting to the recorded one. Blue line, light stimulation.

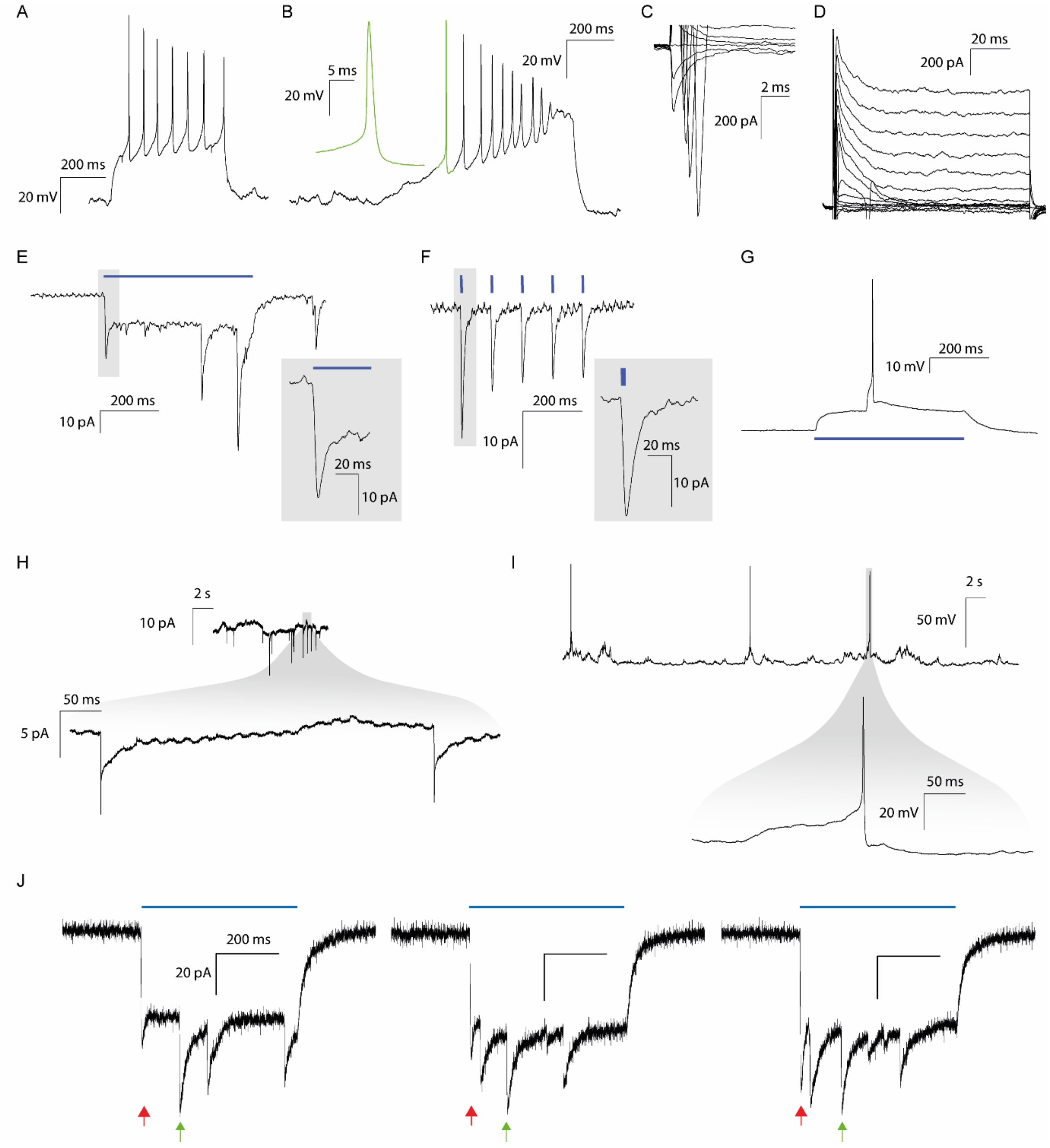

Table S1

Electrophysiological properties of hdINs. The upper table shows the intrinsic electrophysiological properties of hdINs at different time points. The lower table summarizes the properties of the spontaneous synaptic currents of hdINs at the two latest time points. ([a]) Ratio of cells. Mean ± SEM. One-way ANOVA with Tukey's post hoc test for multiple comparisons of means and Fisher's exact test for comparison of proportions (25 DIV n=15, 35 DIV n=36, and 49 DIV n=26) in the upper table. Mann-Whitney test for comparison of medians (35 DIV n=11 and 49 DIV n=8) and Fisher's exact test for comparison of proportions (35 DIV n=26 and 49 DIV n=25) in the bottom table. *, p<.05; **, p<.01; ***, p<.001; ****, p<.0001; indicates a statistically significant difference in comparison to the 25 DIV group in the top table and to the 35 DIV group in the bottom one. AP, action potential; RMP, resting membrane potential; Ri, input resistance.

Intrinsic electrophysiological properties

	hESC-derived Neurons		
	25 DIV	35 DIV	49 DIV
Spontaneous APs [a]	2/15	25/36 ****	21/26 ****
RMP (mV)	**-34.27** ± 2.87	**-47.20** ± 1.84 ****	**-44.85** ± 1.94 ***
R_i (MOhm)	**1470** ± 244.67	**1296.13** ± 178.03	**1205.86** ± 158.35
Fire to Ramps [a]	9/13	35/36 *	25/26 *
Action Potential			
Threshold (mV)	**-34.62** ± 0.94	**-36.39** ± 0.95	**-33.90** ± 1.49
Amplitude (mV)	**51.98** ± 4.36	**75.87** ± 1.7 ****	**75.27** ± 2.62 ****
Duration (ms)	**4.58** ± 0.85	**2.85** ± 0.27 *	**2.14** ± 0.26 ***
AHP amplitude (mV)	**17.58** ± 2.54	**25.16** ± 1.15 **	**28.28** ± 1.23 ***
Max. number	**2** ± 0.73	**17.9** ± 1.80 ****	**18.64** ± 2.39 ****

Electrophysiological properties of spontaneous synaptic currents

	hESC-derived Neurons	
	35 DIV	49 DIV
Spontaneous synaptic activity [a]	13/26	23/25 **
Amplitude (pA)	**34.33** ± 5.82	**49.63** ± 7.39
Inter-event interval, IEI (ms)	**1688.46** ± 322.5	**711.02** ± 107.02
Rising time (ms)	**1.1** ± 0.04	**1.06** ± 0.05
Decay time (ms)	**14.87** ± 0.55	**15.1** ± 0.55

Table S2

Primary antibodies used for immunocytochemistry. Antibodies used for immunocytochemistry detection of different markers for cellular characterization.

Antibody	Host species	Dilution	Company
Oct4	Rabbit	1:500	Abcam ab19857
MAP2	Chicken	1:2000	Abcam ab5392
MAP2	Mouse	1:500	Sigma Aldrich M2320
mCherry	Chicken	1:2000	Abcam ab205402
GABA	Rabbit	1:2000	Sigma Aldrich A2052
GAD65/67	Rabbit	1:500	Sigma Aldrich G5163
Calretinin (CR)	Rabbit	1:1000	Swant CR7697
Calbindin (CB)	Rabbit	1:1000	Swant CB-38a
PV	Mouse	1:1000	Swant PV235
SST	Rat	1:100	Millipore MAB354
NPY	Rabbit	1:5000	Sigma Aldrich N9528
CCK	Rabbit	1:1000	Sigma Aldrich C2581
GFAP	Mouse	1:150	Sigma Aldrich G3793
TH	Mouse	1:200	Millipore MAB318
vGlut1	Mouse	1:200	Synaptic Systems 135511
KGA	Rabbit	1:1000	Abcam ab93434

Table S3

Primer sequences used for gene expression analysis. List of primer sequences used for RT-qPCR detection of specific genes for cellular characterization.

TARGET GENE	PRIMERS	
	**Forward**	**Reverse**
h-*ACTB*	CCTTGCACATGCCGGAG	GCACAGAGCCTCGCCTT
h-*GAPDH*	TTGAGGTCAATGAAGGGGTC	GAAGGTGAAGGTCGGAGTCA
h-*POU5F1* (OCT4)	TCTCCAGGTTGCCTCTCACT	GTGGAGGAAGCTGACAACAA
h-*SOX2*	CATGGCAATCAAAATGTCCA	TTTCACGTTTGCAACTGTCC
h-*MAP2*	CCGTGTGGACCATGGGGCTG	GTCGTCGGGGTGATGCCACG
h-*SYN1*	CCCGTGGTTGTGAAGATGGGGC	TGCCACGACACTTGCGATGTCC
h-*NKX2.1*	AGGGCGGGGCACAGATTGGA	GCTGGCAGAGTGTGCCCAGA
h-*GAD1* (GAD67)	GCCAGACAAGCAGTATGATGT	CCAGTTCCAGGCATTTGTTGAT
h-*PVALB*	TGCAGGATGTCGATGACAGA	TTTCTTCAGGCCGACCATTT
h-*SST*	CCCCAGACTCCGTCAGTTTCT	CATTCTCCGTCTGGTTGGGT
h-*CALB1* (CB)	TGGATCAGTATGGGCAAAGAGA	ATCGGAAGAGCAGCAGGAAAT
h- *CALB2* (CR)	TGGAAGCACTTTGACGCAGAC	CAGAGCCTTTCCTTGCCTTCT
h-*VIP*	TCTCACAGACTTCGGCATGG	TCATTTGCTCCCTCAAAGGGT
h-*NPY*	TGTTCCCAGAACTCGGCTTG	TGCATTGGTAGGATGGGTGG
h-*CCK*	AGGGTATCGCAGAGAACGGA	CTTATCCTGTGGCTGGGGTC
h-*PPP1R1B* (DARPP32)	GAGAGCCTCAGGAGAGGGGCAC	AGGTGGTGTGTAGGCACAGGGG
h-*SLC17A7* (VGLUT1)	AATAACAGCACGACCCACCGCG	AGCCGTGTATGAGGCCGACAGT
h-*GFAP*	AGATCCGCACGCAGTATGA	AGTCGTTGGCTTCGTGCTT
h-*PDGFRA*	CCTTGGTGGCACCCCTTAC	TCCGGTACCCACTCTTGATCTT
m-*Actb*	CATTGCTGACAGGATGCAGAAGG	TGCTGGAAGGTGGACAGTGAGG
m-*Gapdh*	AACTTTGGCATTGTGGAAGG	ACACATTGGGGGTAGGAACA
m-*Gfap*	ACCAGCTTACGGCCAACAGT	TACGCAGCCAGGTTGTTCTC
WPRE	GTCCTTTCCATGGCTGCTC	CCGAAGGGACGTAGCAGA
